# Special Issue on Enabling Technology in Optical Fiber Communications: From Device, System to Networking

**DOI:** 10.3390/s21061969

**Published:** 2021-03-11

**Authors:** Yang Yue, Jian Zhao, Jiangbing Du, Zhaohui Li

**Affiliations:** 1 Institute of Modern Optics, Nankai University, Tianjin 300350, China; 2 School of Precision Instruments and Optoelectronics Engineering, Tianjin University, Tianjin 300072, China; enzhaojian@tju.edu.cn; 3 State Key Laboratory of Advanced Optical Communication Systems and Networks, Shanghai Jiao Tong University, Shanghai 200240, China; dujiangbing@sjtu.edu.cn; 4 State Key Laboratory of Optoelectronic Materials and Technologies, School of Electronics and Information Technology, Sun Yat Sen University, Guangzhou 510275, China; lzhh88@mail.sysu.edu.cn

It is well known that optical fiber communications support the global communication networks nowadays, which originates from Charles K. Kao’s proposal of using optical fiber as a light transmission medium in 1966 [1]. By utilizing different degrees of freedom of the photon, society has made tremendous progress over the past half century. Revolutionary technologies debut one after another, including wavelength-division multiplexing (WDM), coherent detection, space-division multiplexing (SDM), and so forth [2,3]. Recently, the emerging 5G, cloud computing, and high-definition video have been driving more bandwidth and power-hungry applications. To better serve these needs, the optical fiber communications community has been escalating the research and development efforts in device, system, and networking to the next level.

This Special Issue aims to explore the enabling technology in optical fiber communications. It focuses on the state-of-the-art advances from fundamental theories, devices, and subsystems, to networking applications, as well as future perspectives of optical fiber communications. The collected papers have well accomplished these goals by contributing leading-edge derivation, analysis, and experiments with significant results. The topics cover integrated photonics, fiber optics, fiber and free-space optical communications, and optical networking. The special issue consists of one review paper, nine research articles, and eight letters.

More specifically, from the integrated device perspective, Fang, Y. et al. proposed an Si_3_N_4_/SiO_2_ horizontal-slot-waveguide-based polarization beam splitter (PBS) [4]. Its coupling length can be effectively reduced due to the slot design, and the extinction ratios (ER) of the fundamental modes for two orthogonal polarizations are both >20 dB. Furthermore, it features low nonlinearity, which is critical for on-chip high-power systems. For fiber-based devices, Rong, J. et al. numerically simulated shaping the supercontinuum (SC) using the fiber cascading method to significantly increase the SC spectral width and flatness in silica photonic crystal fiber (PCF) [5]. To characterize linear frequency-modulated continuous-wave (FMCW) lasers, Yang, J. et al. [6] proposed a scheme for measuring the mapping relationship between instantaneous frequency and time of a FMCW laser based on a modified coherent optical spectrum analyzer (COSA) and digital signal processing (DSP) method. The authors demonstrated precisely measuring an FMCW laser at a large fast sweep rate of 5000 THz/s, while maintaining <100 kHz uncertainty.

Regarding the system-level technologies for optical communication, Tseng, S. et al. developed a bipolar optical code division multiple access (Bi-OCDMA) technique based on spectral amplitude coding for the formation and transmission of optical-polarized and coded signals over wireless optical channels [7]. The proposed free-space optics communication system used a dual electro-optical modulator design, which could improve the transmission rate. For direct-detection optical communication systems, Zhang, W. et al. compared the complexity, efficiency, and stability performance of pruned Volterra series-based equalization (VE) and neural network-based equalization (NNE) for 112 Gbps vertical-cavity surface-emitting laser (VCSEL)-enabled optical interconnects [8]. The experimental results showed that NNE has more than one order of magnitude bit error rate (BER) advantage over VE at the same computation complexity, and pruned NNE has around 50% lower computation complexity compared to VE at the same BER level. For coherent-detection optical communication systems, Ding, J. et al. investigated the impact of equalization-enhanced phase noise (EEPN) in Nyquist-spaced dual-polarization quadrature amplitude modulation (DP-QAM) links [9]. It was found that EEPN-induced distortions become more significant with the increase of the local oscillator (LO) laser linewidth, and this results in degradations in BER, achievable information rate (AIR), and AIR-distance product. Moreover, Wu, B. et al. proposed a blind discrete-cosine-transform-based phase noise compensation (BD-PNC) to compensate the intercarrier interference (ICI) in the coherent optical offset-quadrature amplitude modulation (OQAM)-based filter-bank multicarrier (CO-FBMC/OQAM) transmission system [10]. The simulation results showed that its BER performance is improved by more than one order of magnitude through the mitigation of the ICI over traditional blind PNC scheme only aiming for common phase error (CPE) compensation. For quantum communication applications, Wu, B. et al. proposed and experimentally demonstrated a secure key generation and distribution system that is compatible with optical amplifiers and standard WDM transmission systems [11]. The key generation system was tested in a 240 km bidirectional fiber-pair link with multiple optical amplifiers, and 38 WDM channels were transmitted together with the key distribution channel.

On the networking level, Holik, M. et al. created an open-source software-based solution for monitoring traffic transmitted through gigabit passive optical network (GPON) [12]. The work described the issue of writing to the Mongo database system, showing that the high processing speed is too high for Python processing and critical operations must be implemented in the C# programming language. He, C. et al. proposed a FiWi broadband access network, integrating the wireless mesh network (WMN) frontend subnetwork, together with time and wavelength division multiplexed PON (TWDM-PON) optical backhaul and adapting power over fiber (PoF) technology [13]. For elastic optical networks (EONs), He, S. et al. proposed an advanced-reservation-based invalid-spectrum-aware (AR-ISA) resource allocation algorithm to improve the networking performance and the resource alignment [14]. Moreover, Rodrigues, E. et al. proposed a crosstalk-aware routing, modulation, spectrum, and core allocation (RMSCA) algorithm that uses a multipath and mapping scheme for improving resource allocation [15]. Simulation results show that the algorithm decreases the blocking ratio by up to four orders of magnitude compared with the other RMSCA algorithms in the literature. Furthermore, Zong, Y. et al. surveyed the state-of-the-art works for the virtual network embedding (VNE) problem towards multidomain heterogeneous converged optical networks, and discussed the future research issues and challenges [16].

Additionally, there are several collected papers aimed at sensing applications. Wu, Z. et al. demonstrated Bragg-grating-assisted Sagnac interferometer in SiO_2_-Al_2_O_3_-La_2_O_3_ polarization-maintaining fiber for strain–temperature discrimination [17]. Xu, X. et al. proposed and demonstrated a temperature and humidity sensor based on a fluorinated polyimide film and fiber Bragg grating [18]. Han, H. et al. proposed a surface plasmon resonance (SPR) sensor based on a dual-side polished microstructured optical fiber (MOF) with a dual core [19]. Wu, Y. et al. proposed a resolution enhancement and signal-to-noise ratio (SNR) improvement scheme for digital optical frequency comb (DOFC)-based Brillouin optical time-domain analysis (BOTDA) ultrafast distributed sensing employing a pump pulse array [20]. Cheng, Y. et al. analyzed the source of the position deviation and proposed a demodulation recursive compensation algorithm to ensure a submillimeter resolution in improved optical frequency domain reflectometry (OFDR) [21].

It has just been over 50 years since the discovery of optical fiber as a low-loss light transmission medium by Charles K. Kao and his coworkers. Within this fairly short period of time, an extensive research community and industry have been established globally. Nowadays, optical fiber communications is the backbone of our information technology infrastructure, supporting voice, video, and data transmission through global networks. One critical issue in its research and development is the challenge of meeting the needs of increasing the data capacity without compromising size, weight, power, and cost (SWaP-C) constraints. Especially during the past decade, photonics integration and coherent detection technologies were booming tremendously. From this trend, we are expecting the co-packaged optics (CPO) to dominate the next-generation optical fiber communication systems. Hopefully, more and more novel technologies, such as SDM, can be commercialized in the near future, to enable new growth for the industry and serve the ever-growing data-traffic demand from society.
